# 2-Thienylcarbonylmethylene–triphenylphosphorane ylide

**DOI:** 10.1107/S1600536808005497

**Published:** 2008-03-05

**Authors:** Seyyed Javad Sabounchei, Vida Jodaian, Hamid Reza Khavasi

**Affiliations:** a Faculty of Chemistry, Bu-Ali Sina University, Hamadan 65174, Iran; b Department of Chemistry, Shahid Beheshti University, Evin, Tehran 1983963113, Iran

## Abstract

In the mol­ecule of the title compound, (2-thienylcarbon­yl)(triphenyl­phospho­nio)methanide, C_24_H_19_OPS, the geometry around the P atom is nearly tetra­hedral and the O—C—C—P torsion angle is 2.80 (3)°. The thio­phene ring is twisted through an angle of 4.33 (4)° with respect to the plane of the carbonyl group. Inter- and intra­molecular hydrogen bonds and C—H⋯π inter­actions are present in the crystal structure.

## Related literature

For related literature, see: Allen *et al.* (1987 ▶); Bart (1969 ▶); Dunitz (1979 ▶).
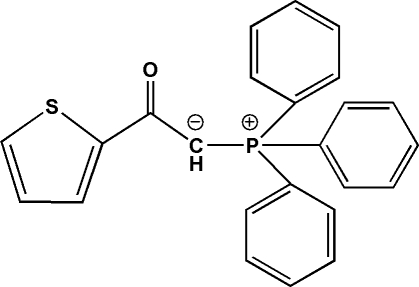


## Experimental

### 

#### Crystal data

C_24_H_19_OPS
*M* *_r_* = 386.43
Monoclinic, 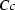

*a* = 11.3076 (17) Å
*b* = 15.474 (2) Å
*c* = 11.3540 (16) Å
β = 97.063 (12)°
*V* = 1971.6 (5) Å^3^
*Z* = 4
Mo *K*α radiation
μ = 0.26 mm^−1^
*T* = 120 (2) K
0.4 × 0.25 × 0.2 mm

#### Data collection

Stoe IPDSII diffractometer
Absorption correction: numerical (*X-RED32*; Stoe & Cie, 2005 ▶) *T*_min_ = 0.930, *T*_max_ = 0.950
7138 measured reflections
4130 independent reflections
4082 reflections with *I* > 2σ(*I*)
*R*_int_ = 0.059

#### Refinement

*R*[*F* ^2^ > 2σ(*F* ^2^)] = 0.042
*wR*(*F* ^2^) = 0.116
*S* = 1.03
4130 reflections
244 parameters
2 restraints
H-atom parameters constrained
Δρ_max_ = 0.73 e Å^−3^
Δρ_min_ = −0.48 e Å^−3^
Absolute structure: Flack (1983 ▶), 3361 Friedel pairs
Flack parameter: 0.03 (7)

### 

Data collection: *X-AREA* (Stoe & Cie, 2005 ▶); cell refinement: *X-AREA*; data reduction: *X-RED32* (Stoe & Cie, 2005 ▶); program(s) used to solve structure: *SHELXS97* (Sheldrick, 2008 ▶); program(s) used to refine structure: *SHELXL97* (Sheldrick, 2008 ▶); molecular graphics: *ORTEP-3 for Windows* (Farrugia, 1997 ▶); software used to prepare material for publication: *WinGX* (Farrugia, 1999 ▶).

## Supplementary Material

Crystal structure: contains datablocks global, I. DOI: 10.1107/S1600536808005497/bq2064sup1.cif

Structure factors: contains datablocks I. DOI: 10.1107/S1600536808005497/bq2064Isup2.hkl

Additional supplementary materials:  crystallographic information; 3D view; checkCIF report

## Figures and Tables

**Table:** 

C6—P1	1.727 (2)
C7—P1	1.806 (2)
C13—P1	1.812 (2)
C19—P1	1.816 (2)

**Table:** 

C6—P1—C7	106.06 (10)
C6—P1—C13	117.09 (12)
C7—P1—C13	106.16 (10)
C6—P1—C19	112.72 (10)
C7—P1—C19	108.67 (10)
C13—P1—C19	105.74 (10)

**Table 2:** Hydrogen-bond geometry (Å, °)

*D*—H⋯*A*	*D*—H	H⋯*A*	*D*⋯*A*	*D*—H⋯*A*
C9—H9⋯O1^i^	0.93	2.34	3.147 (3)	145
C14—H14⋯O1	0.93	2.52	3.187 (3)	129
C3—H3⋯*Cg*1^ii^	0.93	2.82	3.582 (3)	140
C8—H8⋯*Cg*2^ii^	0.93	2.80	3.592 (2)	143
C10—H10⋯*Cg*2^i^	0.93	2.95	3.734 (3)	143
C23—H23⋯*Cg*3^iii^	0.93	2.82	3.465 (3)	127

Symmetry code: (i) 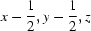; (ii) 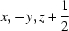; (iii) 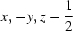. *Cg*1 is the centroid of atoms C7–C12, *Cg*2 is the centroid of atoms C19–C24 and *Cg*3 is the centroid of atoms C13–C18.

**Fig. 1.**
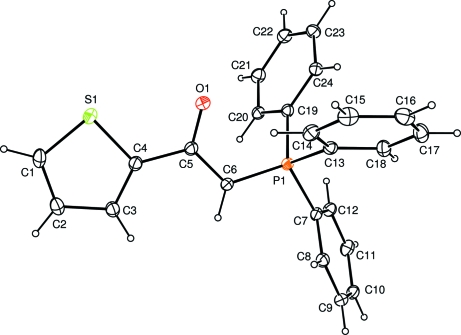
The molecular structure with the atom-numbering scheme. Displacement ellipsoids are drawn at 30% probability level.

**Fig. 2.**
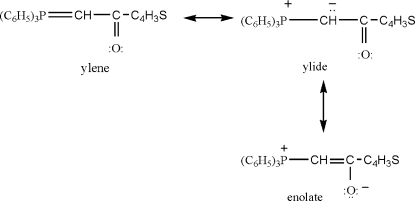
Resonance in the TPPY.

**Fig. 3.**
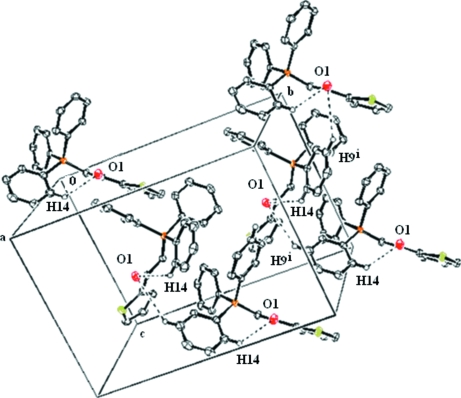
The packing of (I). Hydrogen bonds are shown as dashed lines. H atoms not participate in H bonding are omitted for clarity. Symmetry code: (i) -1/2 + x,-1/2 + y,z.

## supplementary crystallographic information

### Comment

Phosphoranes of the type (C_6_H_5_)_3_PCHCOC_4_H_3_S (TPPY) can coordinate to metals through either C or O atoms. The crystal and molecular structure of this ylide was determined successfully. The structural investigation with metrical parameters for the title compound, (I), show that how they vary with a change in delocalization in the metal derivatives as well as in other resonance stabilized ylides. In this molecule, the bond lengths and angles (Table 1) are generally within normal ranges (Allen *et al.*, 1987).

The P1—C6 bond length [1.727 (2) Å], is shorter than the other P—C bonds (Table 1) and longer than the equivalent bond lengths of 1.66 Å reported for methylenetriphenylphosphorane (Bart, 1969), which shows partial double-bond character for these two bonds.

The C4—S1 and C1—S1 bond lengths of 1.712 (3) Å and 1.719 (2) Å are longer than the other C—C bonds. These bond distances suggest resonance delocalization in the molecule (Fig. 2). The resonance formulation is supported by the near planarity of P1, C6, C5 and O1 in TPPY. The torsion angle O1—C2—C1—P1 of 2.80 (3)° also indicates resonance.

The thiophenyl group is twisted with respect to the plane containing the carbonyl group through angles of 4.33 (4)°. Bond angle of 118.30 (16)° for P1—C6—C5, indicate a distorted trigonal arrangement about C6. The non-bonded distances P1—O1 of 2.990 (3) Å of TPPY is significantly shorter than the sum of the van der Waals radii of P and O (3.3 Å) (Dunitz, 1979), indicating a strong intramolecular interaction between P^+^ and O^-^ charge centers, which leads to the *cis* orientation. Packing diagram of TPPY is shown in Fig. 3. A s it is clear from this diagram, there are some C—H···O inter- and intra-molecular interactions that seem to be effective in stability of packing (Table 2). There are four remarkable C—H···*Cg* (pi-ring) interactions; [H3···*Cg*1(C7/12)^i^ = 2.82 Å and C3—H3···*Cg*1 = 140°, H8···*Cg*2(C19/24)^i^ = 2.80 Å and C8—H8···*Cg*2 = 143°, H10···*Cg*2(C19/24)^ii^ = 2.95 Å and C10—H10···*Cg*2 = 143°, H23···*Cg*3(C13/18)^iii^ = 2.82 Å and C23—H23···*Cg*1 = 127°, with symmetry codes; (i) *X*,-Y,1/2+*Z*, (ii) -1/2+*X*,-1/2+Y,*Z* and (iii) *X*,-Y,-1/2+*Z*] which are effective in the stabilization of crystal packing.

### Experimental

The title compound was prepared by addition of 2-bromo-acetothiophen (0.102 g, 0.5 mmol) in chloroform (25 ml) to a solution of triphenylphosphine (0.131 g, 0.5 mmol) in the same solvent (5 ml). The resulting pale pink solution was stirred for 12 h. The solution was concentrated under reduced pressure to 5 ml, and diethyl ether (20 ml) was added. The yellow solid formed was filtered off, washed with petroleum diethyl ether (10 ml), and dried under reduced pressure. In order to get the final product, all of the crude solid was transferred to an alkaline solution of 5% NaOH and stirred at 310 K for about 14 h, yielding the white precipitate. The product was washed several times with distilled water and air dried. The resulting solid was recrystallized from a chloroform-diethyl ether mixture (m.p. 496–498 K). Yield: 78%, 0.301 g.

### Refinement

H atoms were positioned geometrically, with C—H=0.93 Å for aromatic and methine H and constrained to ride on their parent atoms with *U*_iso_(H)=1.2*U*_eq_(C).

### Crystal data

**Table:**

C_24_H_19_O_1_P_1_S_1_	*F*_000_ = 808
*M**_r_* = 386.43	*D*_x_ = 1.302 Mg m^−^^3^
Monoclinic, *C**c*	Mo *K*α radiation λ = 0.71073 Å
Hall symbol: C -2yc	Cell parameters from 2000 reflections
*a* = 11.3076 (17) Å	θ = 2.2–27.9º
*b* = 15.474 (2) Å	µ = 0.26 mm^−^^1^
*c* = 11.3540 (16) Å	*T* = 120 (2) K
β = 97.063 (12)º	Prism, colorless
*V* = 1971.6 (5) Å^3^	0.4 × 0.25 × 0.2 mm
*Z* = 4	

### Data collection

**Table:**

Stoe IPDSII diffractometer	*R*_int_ = 0.059
rotation method scans	θ_max_ = 25.9º
Absorption correction: numerical(X-RED32; Stoe & Cie, 2005)	θ_min_ = 2.2º
*T*_min_ = 0.930, *T*_max_ = 0.950	*h* = −14→14
7138 measured reflections	*k* = −20→20
4130 independent reflections	*l* = −14→14
4082 reflections with *I* > 2σ(*I*)	

### Refinement

**Table:**

Refinement on *F*^2^	H-atom parameters constrained
Least-squares matrix: full	*w* = 1/[σ^2^(*F*_o_^2^) + (0.0801*P*)^2^ + 1.5059*P*] where *P* = (*F*_o_^2^ + 2*F*_c_^2^)/3
*R*[*F*^2^ > 2σ(*F*^2^)] = 0.042	(Δ/σ)_max_ = 0.007
*wR*(*F*^2^) = 0.116	Δρ_max_ = 0.74 e Å^−^^3^
*S* = 1.03	Δρ_min_ = −0.48 e Å^−^^3^
4130 reflections	Extinction correction: none
244 parameters	Absolute structure: Flack (1983), 3361 Friedel pairs
2 restraints	Flack parameter: 0.03 (7)

### Special details

**Table:**

Geometry. All e.s.d.'s (except the e.s.d. in the dihedral angle between two l.s. planes) are estimated using the full covariance matrix. The cell e.s.d.'s are taken into account individually in the estimation of e.s.d.'s in distances, angles and torsion angles; correlations between e.s.d.'s in cell parameters are only used when they are defined by crystal symmetry. An approximate (isotropic) treatment of cell e.s.d.'s is used for estimating e.s.d.'s involving l.s. planes.

### Fractional atomic coordinates and isotropic or equivalent isotropic displacement parameters (Å^2^)

**Table:**

	*x*	*y*	*z*	*U*_iso_*/*U*_eq_	
C1	0.0560 (3)	0.37507 (16)	0.7865 (2)	0.0305 (5)	
H1	0.0667	0.4255	0.8311	0.037*	
C2	−0.0358 (2)	0.31826 (17)	0.7929 (2)	0.0297 (5)	
H2	−0.0942	0.3254	0.8431	0.036*	
C3	−0.0319 (2)	0.24653 (15)	0.7135 (2)	0.0262 (5)	
H3	−0.0875	0.2019	0.7058	0.031*	
C4	0.0640 (2)	0.25175 (14)	0.6502 (2)	0.0232 (4)	
C5	0.1026 (2)	0.19364 (13)	0.5570 (2)	0.0224 (4)	
C6	0.0300 (2)	0.12304 (14)	0.5188 (2)	0.0221 (4)	
H6	−0.0331	0.1068	0.5593	0.026*	
C7	−0.0633 (2)	−0.00552 (13)	0.3568 (2)	0.0208 (4)	
C8	−0.0838 (2)	−0.06819 (14)	0.4413 (2)	0.0243 (5)	
H8	−0.0315	−0.0733	0.5109	0.029*	
C9	−0.1822 (2)	−0.12279 (15)	0.4217 (2)	0.0272 (5)	
H9	−0.196	−0.1638	0.4783	0.033*	
C10	−0.2600 (2)	−0.11563 (15)	0.3166 (2)	0.0274 (5)	
H10	−0.3262	−0.1516	0.3034	0.033*	
C11	−0.2385 (2)	−0.05476 (15)	0.2316 (2)	0.0295 (5)	
H11	−0.29	−0.0507	0.1613	0.035*	
C12	−0.1407 (2)	0.00010 (14)	0.2511 (2)	0.0240 (4)	
H12	−0.1267	0.0406	0.1939	0.029*	
C13	0.1935 (2)	0.00050 (14)	0.4095 (2)	0.0212 (4)	
C14	0.2881 (2)	0.01748 (15)	0.4970 (2)	0.0277 (5)	
H14	0.2825	0.0619	0.5511	0.033*	
C15	0.3913 (3)	−0.03241 (18)	0.5029 (3)	0.0340 (5)	
H15	0.4547	−0.0212	0.5611	0.041*	
C16	0.3994 (2)	−0.09902 (18)	0.4220 (3)	0.0345 (6)	
H16	0.4685	−0.1321	0.4259	0.041*	
C17	0.3045 (3)	−0.11623 (17)	0.3350 (2)	0.0327 (6)	
H17	0.3102	−0.1607	0.281	0.039*	
C18	0.2014 (3)	−0.06698 (14)	0.3290 (2)	0.0272 (5)	
H18	0.1376	−0.0789	0.2714	0.033*	
C19	0.0732 (2)	0.13744 (14)	0.26848 (19)	0.0210 (4)	
C20	−0.0162 (2)	0.19924 (14)	0.2397 (2)	0.0233 (4)	
H20	−0.0793	0.2037	0.2847	0.028*	
C21	−0.0099 (2)	0.25406 (15)	0.1430 (2)	0.0280 (5)	
H21	−0.0703	0.294	0.1219	0.034*	
C22	0.0866 (3)	0.24901 (15)	0.0782 (2)	0.0284 (5)	
H22	0.0913	0.2864	0.0149	0.034*	
C23	0.1750 (2)	0.18886 (16)	0.1075 (2)	0.0269 (5)	
H23	0.2394	0.1859	0.064	0.032*	
C24	0.1684 (2)	0.13204 (15)	0.2025 (2)	0.0238 (4)	
H24	0.2276	0.0908	0.2213	0.029*	
O1	0.19781 (17)	0.21153 (10)	0.51549 (16)	0.0280 (4)	
P1	0.06067 (5)	0.06711 (3)	0.39443 (5)	0.01906 (13)	
S1	0.14871 (6)	0.34234 (4)	0.68674 (6)	0.02845 (15)	

### Atomic displacement parameters (Å^2^)

**Table:**

	*U*^11^	*U*^22^	*U*^33^	*U*^12^	*U*^13^	*U*^23^
C1	0.0429 (15)	0.0251 (10)	0.0223 (11)	0.0022 (10)	−0.0003 (10)	−0.0063 (8)
C2	0.0360 (14)	0.0310 (12)	0.0225 (11)	0.0044 (10)	0.0055 (10)	−0.0033 (9)
C3	0.0328 (13)	0.0238 (10)	0.0224 (10)	0.0021 (9)	0.0053 (9)	−0.0005 (8)
C4	0.0281 (11)	0.0195 (9)	0.0218 (10)	−0.0002 (8)	0.0028 (9)	−0.0007 (7)
C5	0.0286 (11)	0.0182 (9)	0.0208 (10)	−0.0013 (8)	0.0042 (9)	0.0008 (7)
C6	0.0279 (12)	0.0186 (9)	0.0205 (10)	−0.0044 (8)	0.0063 (9)	−0.0017 (7)
C7	0.0244 (10)	0.0163 (9)	0.0218 (10)	−0.0007 (7)	0.0032 (8)	−0.0018 (7)
C8	0.0301 (13)	0.0212 (10)	0.0220 (11)	0.0002 (8)	0.0044 (9)	0.0001 (7)
C9	0.0341 (13)	0.0218 (10)	0.0274 (11)	−0.0056 (9)	0.0105 (10)	−0.0016 (8)
C10	0.0248 (11)	0.0218 (10)	0.0365 (13)	−0.0043 (8)	0.0074 (10)	−0.0063 (9)
C11	0.0287 (13)	0.0251 (10)	0.0331 (12)	0.0011 (9)	−0.0029 (10)	−0.0026 (9)
C12	0.0261 (12)	0.0206 (10)	0.0246 (10)	0.0003 (8)	0.0006 (9)	0.0015 (8)
C13	0.0229 (11)	0.0192 (9)	0.0218 (10)	0.0000 (8)	0.0037 (8)	0.0036 (8)
C14	0.0293 (13)	0.0253 (10)	0.0276 (11)	−0.0017 (9)	−0.0001 (10)	0.0018 (9)
C15	0.0291 (13)	0.0361 (13)	0.0357 (13)	0.0024 (10)	−0.0001 (11)	0.0084 (11)
C16	0.0282 (13)	0.0358 (13)	0.0407 (14)	0.0092 (10)	0.0097 (11)	0.0125 (11)
C17	0.0407 (15)	0.0290 (11)	0.0294 (12)	0.0107 (10)	0.0075 (11)	0.0021 (9)
C18	0.0329 (14)	0.0247 (12)	0.0243 (12)	0.0048 (9)	0.0047 (10)	0.0001 (8)
C19	0.0252 (11)	0.0192 (9)	0.0186 (10)	−0.0005 (8)	0.0024 (8)	−0.0009 (7)
C20	0.0282 (12)	0.0178 (9)	0.0243 (10)	0.0011 (8)	0.0051 (9)	0.0022 (7)
C21	0.0336 (13)	0.0234 (10)	0.0263 (11)	0.0018 (9)	0.0011 (10)	0.0045 (8)
C22	0.0384 (14)	0.0265 (11)	0.0201 (10)	−0.0044 (10)	0.0020 (10)	0.0042 (8)
C23	0.0323 (12)	0.0293 (11)	0.0200 (10)	−0.0031 (9)	0.0064 (9)	0.0007 (8)
C24	0.0270 (12)	0.0225 (9)	0.0226 (11)	−0.0011 (8)	0.0054 (9)	0.0004 (8)
O1	0.0299 (9)	0.0235 (8)	0.0325 (9)	−0.0059 (6)	0.0108 (8)	−0.0035 (6)
P1	0.0227 (3)	0.0161 (2)	0.0186 (2)	−0.00069 (19)	0.00337 (19)	0.00047 (18)
S1	0.0342 (3)	0.0220 (3)	0.0294 (3)	−0.0054 (2)	0.0047 (2)	−0.0052 (2)

### Geometric parameters (Å, °)

**Table:**

C1—C2	1.369 (4)	C13—C14	1.392 (4)
C1—S1	1.712 (3)	C13—C18	1.398 (3)
C1—H1	0.93	C13—P1	1.812 (2)
C2—C3	1.434 (3)	C14—C15	1.394 (4)
C2—H2	0.93	C14—H14	0.93
C3—C4	1.375 (3)	C15—C16	1.392 (4)
C3—H3	0.93	C15—H15	0.93
C4—C5	1.494 (3)	C16—C17	1.392 (4)
C4—S1	1.719 (2)	C16—H16	0.93
C5—O1	1.258 (3)	C17—C18	1.388 (4)
C5—C6	1.403 (3)	C17—H17	0.93
C6—P1	1.727 (2)	C18—H18	0.93
C6—H6	0.93	C19—C24	1.388 (3)
C7—C12	1.399 (3)	C19—C20	1.401 (3)
C7—C8	1.403 (3)	C19—P1	1.816 (2)
C7—P1	1.806 (2)	C20—C21	1.396 (3)
C8—C9	1.393 (4)	C20—H20	0.93
C8—H8	0.93	C21—C22	1.391 (4)
C9—C10	1.398 (4)	C21—H21	0.93
C9—H9	0.93	C22—C23	1.377 (4)
C10—C11	1.391 (4)	C22—H22	0.93
C10—H10	0.93	C23—C24	1.401 (3)
C11—C12	1.391 (3)	C23—H23	0.93
C11—H11	0.93	C24—H24	0.93
C12—H12	0.93		
			
C2—C1—S1	112.04 (18)	C13—C14—H14	120.1
C2—C1—H1	124	C15—C14—H14	120.1
S1—C1—H1	124	C16—C15—C14	120.1 (3)
C1—C2—C3	112.4 (2)	C16—C15—H15	120
C1—C2—H2	123.8	C14—C15—H15	120
C3—C2—H2	123.8	C17—C16—C15	120.1 (2)
C4—C3—C2	111.7 (2)	C17—C16—H16	119.9
C4—C3—H3	124.1	C15—C16—H16	119.9
C2—C3—H3	124.1	C18—C17—C16	120.0 (2)
C3—C4—C5	130.7 (2)	C18—C17—H17	120
C3—C4—S1	111.94 (17)	C16—C17—H17	120
C5—C4—S1	117.31 (17)	C17—C18—C13	119.9 (3)
O1—C5—C6	123.4 (2)	C17—C18—H18	120.1
O1—C5—C4	118.1 (2)	C13—C18—H18	120.1
C6—C5—C4	118.5 (2)	C24—C19—C20	120.0 (2)
C5—C6—P1	118.30 (16)	C24—C19—P1	121.98 (18)
C5—C6—H6	120.9	C20—C19—P1	117.98 (16)
P1—C6—H6	120.9	C21—C20—C19	119.6 (2)
C12—C7—C8	119.4 (2)	C21—C20—H20	120.2
C12—C7—P1	123.35 (16)	C19—C20—H20	120.2
C8—C7—P1	117.22 (19)	C22—C21—C20	120.0 (2)
C9—C8—C7	120.4 (2)	C22—C21—H21	120
C9—C8—H8	119.8	C20—C21—H21	120
C7—C8—H8	119.8	C23—C22—C21	120.3 (2)
C8—C9—C10	119.7 (2)	C23—C22—H22	119.9
C8—C9—H9	120.2	C21—C22—H22	119.9
C10—C9—H9	120.2	C22—C23—C24	120.3 (2)
C11—C10—C9	120.1 (2)	C22—C23—H23	119.9
C11—C10—H10	120	C24—C23—H23	119.9
C9—C10—H10	120	C19—C24—C23	119.7 (2)
C12—C11—C10	120.4 (2)	C19—C24—H24	120.1
C12—C11—H11	119.8	C23—C24—H24	120.1
C10—C11—H11	119.8	C6—P1—C7	106.06 (10)
C11—C12—C7	120.0 (2)	C6—P1—C13	117.09 (12)
C11—C12—H12	120	C7—P1—C13	106.16 (10)
C7—C12—H12	120	C6—P1—C19	112.72 (10)
C14—C13—C18	120.2 (2)	C7—P1—C19	108.67 (10)
C14—C13—P1	121.02 (17)	C13—P1—C19	105.74 (10)
C18—C13—P1	118.8 (2)	C1—S1—C4	91.86 (13)
C13—C14—C15	119.7 (2)		
			
S1—C1—C2—C3	−0.8 (3)	C20—C21—C22—C23	1.3 (4)
C1—C2—C3—C4	0.4 (3)	C21—C22—C23—C24	0.2 (4)
C2—C3—C4—C5	−178.7 (2)	C20—C19—C24—C23	0.3 (3)
C2—C3—C4—S1	0.2 (3)	P1—C19—C24—C23	−178.83 (18)
C3—C4—C5—O1	−177.3 (3)	C22—C23—C24—C19	−1.0 (4)
S1—C4—C5—O1	3.9 (3)	C5—C6—P1—C7	−170.32 (19)
C3—C4—C5—C6	3.8 (4)	C5—C6—P1—C13	71.5 (2)
S1—C4—C5—C6	−175.02 (18)	C5—C6—P1—C19	−51.5 (2)
O1—C5—C6—P1	−9.7 (3)	C12—C7—P1—C6	115.89 (19)
C4—C5—C6—P1	169.12 (17)	C8—C7—P1—C6	−61.6 (2)
C12—C7—C8—C9	−1.7 (3)	C12—C7—P1—C13	−118.89 (19)
P1—C7—C8—C9	175.91 (17)	C8—C7—P1—C13	63.60 (19)
C7—C8—C9—C10	0.7 (3)	C12—C7—P1—C19	−5.6 (2)
C8—C9—C10—C11	0.6 (4)	C8—C7—P1—C19	176.93 (17)
C9—C10—C11—C12	−0.8 (4)	C14—C13—P1—C6	−23.8 (2)
C10—C11—C12—C7	−0.2 (3)	C18—C13—P1—C6	158.29 (17)
C8—C7—C12—C11	1.5 (3)	C14—C13—P1—C7	−141.96 (18)
P1—C7—C12—C11	−176.01 (18)	C18—C13—P1—C7	40.1 (2)
C18—C13—C14—C15	0.8 (3)	C14—C13—P1—C19	102.69 (19)
P1—C13—C14—C15	−177.07 (18)	C18—C13—P1—C19	−75.2 (2)
C13—C14—C15—C16	−0.1 (4)	C24—C19—P1—C6	131.09 (19)
C14—C15—C16—C17	−0.2 (4)	C20—C19—P1—C6	−48.1 (2)
C15—C16—C17—C18	0.0 (4)	C24—C19—P1—C7	−111.6 (2)
C16—C17—C18—C13	0.7 (4)	C20—C19—P1—C7	69.2 (2)
C14—C13—C18—C17	−1.1 (3)	C24—C19—P1—C13	2.0 (2)
P1—C13—C18—C17	176.84 (18)	C20—C19—P1—C13	−177.19 (18)
C24—C19—C20—C21	1.2 (4)	C2—C1—S1—C4	0.7 (2)
P1—C19—C20—C21	−179.61 (18)	C3—C4—S1—C1	−0.5 (2)
C19—C20—C21—C22	−2.0 (4)	C5—C4—S1—C1	178.5 (2)

### Hydrogen-bond geometry (Å, °)

**Table:**

*D*—H···*A*	*D*—H	H···*A*	*D*···*A*	*D*—H···*A*
C9—H9···O1^i^	0.93	2.34	3.147 (3)	145
C14—H14···O1	0.93	2.52	3.187 (3)	129
C3—H3···Cg1^ii^	0.93	2.82	3.582 (3)	140
C8—H8···Cg2^ii^	0.93	2.80	3.592 (2)	143
C10—H10···Cg2^i^	0.93	2.95	3.734 (3)	143
C23—H23···Cg3^iii^	0.93	2.82	3.465 (3)	127

Symmetry codes: (i) *x*−1/2, *y*−1/2, *z*; (ii) *x*, −*y*, *z*+1/2; (iii) *x*, −*y*, *z*−1/2.
